# MicroRNA-186 suppresses cell proliferation and metastasis in bladder cancer

**DOI:** 10.4314/ahs.v22i4.8

**Published:** 2022-12

**Authors:** Jun Feng Liang, Pei hua Li, Yong Zhu, Shuai shuai Zheng, Jing wei Liu, Shi qiang Song

**Affiliations:** Department of Urology, Qingdao Chengyang People's hospital, Qing dao, Shan dong Province, P.R.China

**Keywords:** miR-186, ADAMTS12, bladder cancer, proliferation, metastasis biomarker

## Abstract

**Purpose:**

Bladder cancer (BCa) is a common malignancy in the urinary system. This study aims to explore the role of miR-186 in BCa tumorigenesis.

**Methods:**

The expression of miR-186 and ADAMTS12 in clinical BCa tissues and cell lines was detected. BCa cell lines T24, 5637 and EJ were used to transfect miR-186 mimics or inhibitors. Luciferase reporter gene detection confirmed the correlation between miR-186 and ADAMTS12. MTT method and flow cytometry were used to detect cell viability and apoptosis. Cell migration and invasion ability was detected by transwell assay. The protein level of ADAMTS12, β-catenin, GSK-3β and p-GSK-3β was determined using western blot analysis.

**Results:**

MiR-186 was negatively correlated with the expression of ADAMTS12 in BCa tissues. Further research confirmed that ADAMTS12 is the direct target of miR-186. In addition, overexpression of miR-186 down-regulated the expression of ADAMTS12, inhibiting cell viability and apoptosis, while knockout of miR-186 led to the opposite result. miR-186 also inhibits the phosphorylation of GSK-3 β and β-catenin without changing the total GSK-3β level. Our study shows that miR-186 has a negative regulatory effect on the expression of ADAMTS12 in clinical specimens and in vitro. miR-186 can inhibit the proliferation and invasion of BCa cells.

**Conclusions:**

miR-186 has the potential to be used as a biomarker in the early detection of BCa.

## Introduction

Bladder cancer (BCa) is a common malignancy with the tenth and ninth highest incidence and mortality, respectively, rates worldwide since 2018 in the urinary system^1^. In China, BCa incidence and mortality rates were 4.46 and 1.91, respectively, in 2016 2. The epidemiological data shows that nearly 70%–80% of bladder cancer is diagnosed as non-muscle invasive bladder cancer (NMIBC)^3^–^4^. Though approximately half of the total number of NMIBC is likely to recur and a few of cases may turn into muscle-invasive type (MIBC) 5, the overall survival rate of NMIBC is up to about 90% when diagnosed at early-stage 6. The current most prevalent treatment for NMIBC is transurethral resection. For MIBC, the treatment of cystectomy usually accompanied with intravesical chemotherapy or immunotherapy^7^–^8^. Therefore, the implications of transcriptional molecular mechanism dysregulation in the poor prognosis of MIBC or advanced BCa need to be further elucidated for early diagnosis and treatment. In recent years, accumulating reports have implied that dysregulation of long non-coding RNA (lncRNA) and microRNA (miRNA) subsets, such as DANCR, GAS5, MALAT1, and miR-200s, plays crucial roles in advanced BCa cell aggressiveness and metastasis via the enrichment of messenger RNA (mRNA)^9^. MicroRNAs (miRNAs) are a type of small non-coding RNAs that involve in a series of biological function^10^–^11^. MiRNA dysregulation frequently occurs in BCa development, progression and metastasis 12–13. For example, miR-375-3p blocks the Wnt/β-catenin pathway and inhibits bladder cancer progression 14, miR-502-5p inhibits cell migration and proliferation in bladder cancer 15. Therefore, the key molecules associated with advanced BCa need to be further investigated. MiR-186 was found to be abnormally expressed in a variety of cancers. Specifically, miR-186 is down-regulated in prostate cancer 16, hepatocellular carcinoma 17, and breast cancer 18, but upregulated in lung adenocarcinoma 19 and glioma 20. However, the correlation between miR-186 and BCa has not been focused yet.

In our current study, we explored the effect of miR-186 in BCa, and investigated the probable mechanism of miR-186 in BCa. Our study will help to better understand the underlying molecular mechanism of Bca.

## Materials and methods

### Clinical specimens

This study was conducted under the permission of Institutional Ethics Committee of Qingdao Chengyang People's hospital. 46 bladder cancer tissues and matched normal specimens were collected from surgical patients in the Department of Urology, Qingdao Chengyang People's hospital. The prior written consent is fully informed and signed by all participants. All samples were stored at -80°C until RNA was extracted.

### Cell lines and culture

Three BCa cell lines (T24, EJ and 5637) and one human immortalized uroepithelial cell line (SV-HUC-1) were obtained from the ATCC (American Type Culture Collection, Manassas, Virginia). Three BCa cells were cultured in RPMI 1640 medium, SV-HUC-1 cell line was cultured in F-12K Medium (HyClone, China), both of which were supplemented with 10% fetal bovine serum (GIBCO, MA, USA), at 37°C with 5% CO_2_.

### Cell transfection

Cells were transfected using Lipofectamine 2000 (Invitrogen, Carlsbad, CA) after 6 hours of incubation. MiR-186 mimics, miR-186 inhibitors and their negative controls (NC) were synthesized from Biofavor Biotech company (Wuhan, China). Before reaching the 90% confluence point, the cells were given 24 hours of starvation for further analysis.

The wild-type sequence of the 3′untranslated region (3′ UTR) of ADAMTS12 containing the miR-186 binding site was amplified by PCR. The 3′UTR sequence of mutant strain EZH2 was obtained by overlap extension PCR method. The wild-type and mutant-type sequences were inserted into the psiCHECK-2 vector (Promega, Madison, WI, USA).

### Luciferase Reporter Assays

For luciferase reporter gene detection, cells were co-transfected with miR-186 mimic and ADAMTS12-3′UTR-luciferase plasmid. The luciferase activity was measured by a dual luciferase reporter analysis system (Promega, Madison, WI, USA). Each experiment is in triplicate.

### Western blotting

Total proteins of BCa cells were extracted by RIPA Lysis Buffer, separated by SDS- polyacrylamide gel electrophoresis (SDS-PAGE) and then transferred onto a piece of polyvinylidene difluoride transfer (PVDF) membrane. Subsequently, all cell membranes were incubated with the following primary antibodies against ADAMTS12(ab203102, Abcam, UK), β-catenin (ab32572, Abcam, UK), p-GSK-3β (sc-373800, Santa Cruz, CA) and GSK-3β (sc-377213, Santa Cruz, CA) and GAPDH (sc-47724, Santa Cruz, CA) at 4°C overnight After incubating with the secondary antibody for 1 hour at room temperature, all bands were measured using an ECL system kit (MultiSciences, Hangzhou, China).

### Quantitative real-time PCR

Trizol reagent (InTrogen, CA, USA) was used to extract total RNA from clinical specimens and BC cells. Then, all RNAs were reverse transcribed into cDNA using a reverse transcription kit (Dalian Kaohsiung Biotechnology). Real-time quantitative PCR was performed by applying the Biosystems SYBR Green Hybrid Kit and ABI 7900 real-time PCR system (Applied Biosystems Life Technology, Foster City, CA, USA). Relative miR-186 or ADAMTS12 mRNA expression were normalized to snRNA U6 (for miRNAs) or GAPDH (for mRNAs) respectively.

### Cell Proliferation Assay

The effect of miR-186 on cell viability was measured by CCK-8 method. Cells are cultured in 96-well plates (2 x 103 cells per well) for 24 hours. Cell use 10µ per well (Sigma-Aldrich, Shanghai, China) 5 mg/mL CCK-8, 37°C for 4h°C. Then discard the medium and add 1% dimethyl sulfoxide (DMSO) to 150µ. An ELX-800 spectrometer reader (Bio-Tek Instruments, Winooski, USA) was used to detect the absorbance at 490 nm.

### Cell apoptosis assay

Cells were collected and stained with Annexin V and propidium iodide using the FITC/PI Apoptosis Detection kit (BD Biosciences). The apoptotic rate was analyzed by flow cytometry (BD Biosciences).

### Immunohistochemistry

Immunohistochemical staining was used to detect the expression of ADAMTS12. The tissue was fixed with 4% paraformaldehyde, embedded in paraffin, and sectioned with a 3 µm section. After incubation with primary (ab203102, Abcam, UK) and secondary antibody (ab150077, Abcam, UK), the sections were stained with DAB reagent. All slices were photographed at a magnification of × 400.

### Statistical Analysis

All data are expressed as mean ± standard deviation. GraphPad Prism (Version 8.0) and SPSS 25.0 were used to analyze all the data. The differences between groups were analyzed through Student`s t test and one-way ANOVA. P-value< 0.05 was considered to be statistically significant.

## Results

### ADAMTS12 negatively correlates with the expression of miR-186 in BCa tissues.

**Figure 1:**
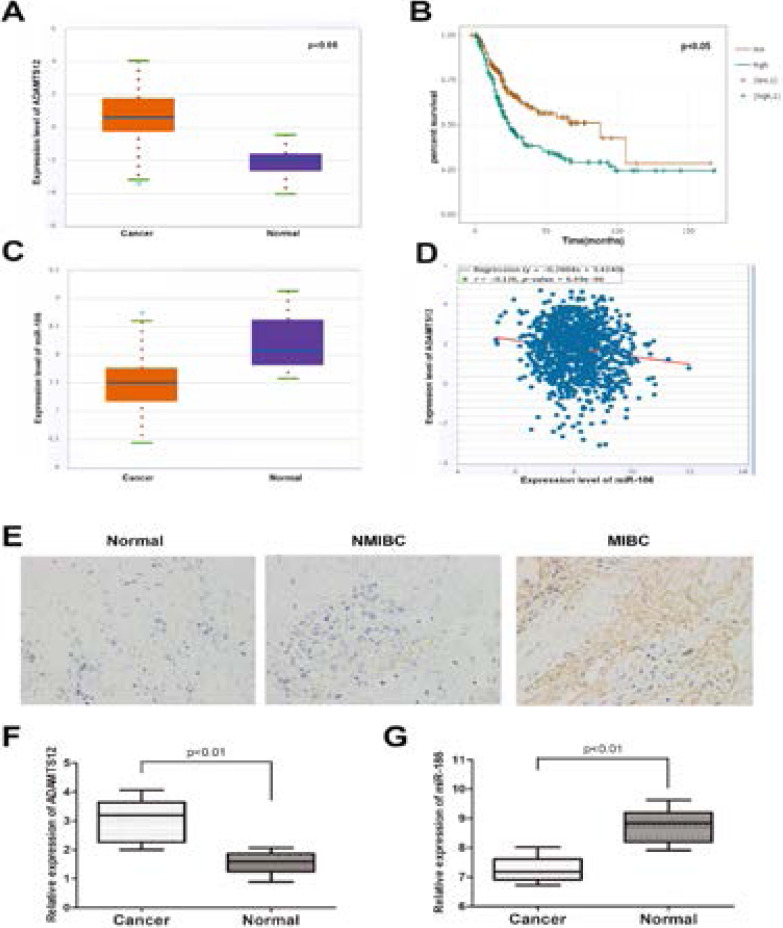
The correlation between miR-186 and ADAMTS12 in BCa samples. (A) Starbase 2.0 data showed that ADAMTS12 was expressed in BCa tissues and paired normal tissues (B) Kaplan-Meier analysis of ADAMTS12 expression and BCa patient total Correlation of survival rate (C) Data from starbase2.0 shows that miR-186 is expressed in BCa tissues and paired normal tissues (D) Data from Starbase 2.0 shows the expression of miR-186 in a two-tailed Pearson correlation analysis It is negatively correlated with the expression of ADAMTS12 (E) Immunohistochemical staining of ADAMTS12 in normal bladder tissue, NMIBC tissue and MIBC tissue (F) Block diagram shows the relative expression of ADAMTS12 in BCa tissue and paired normal tissues (G) Block diagram shows the relative expression of miR-186 in BCa tissues and matched normal tissues.

In order to detect the expression of ADAMTS12 in BCa tissues and normal tissues, we explored the expression of ADAMTS12 from the TCGA data portal of Star base ver2.0. The results showed that compared with normal tissues, ADAMTS12 increased significantly in BCa tissue. In addition, the data showed that the overall survival time of patients with high ADAMTS12 expression was worse than that of patients with low ADMTS12 levels (Figure 1B). Then, we measured the expression of ADAMTS12 in NMIBC, MIBC and normal tissues. Comparing to NMIBC tissues, the expression of ADAMTS12 significantly increased in MIBC tissues (Fig. 1E). Meanwhile, database revealed a lower expression of miR-186 in BC samples than in paired normal samples (Fig. 1C). QRTPCR verified a same expression tendency in clinical samples collected from our department (Fig. 1F, G). A Pearson's correlation analysis revealed that the expression of miR-186 was negatively correlated with ADAMTS12 expression (Fig. 1D).

### ADAMTS12 serves as a direct target of miR-186

**Figure 2:**
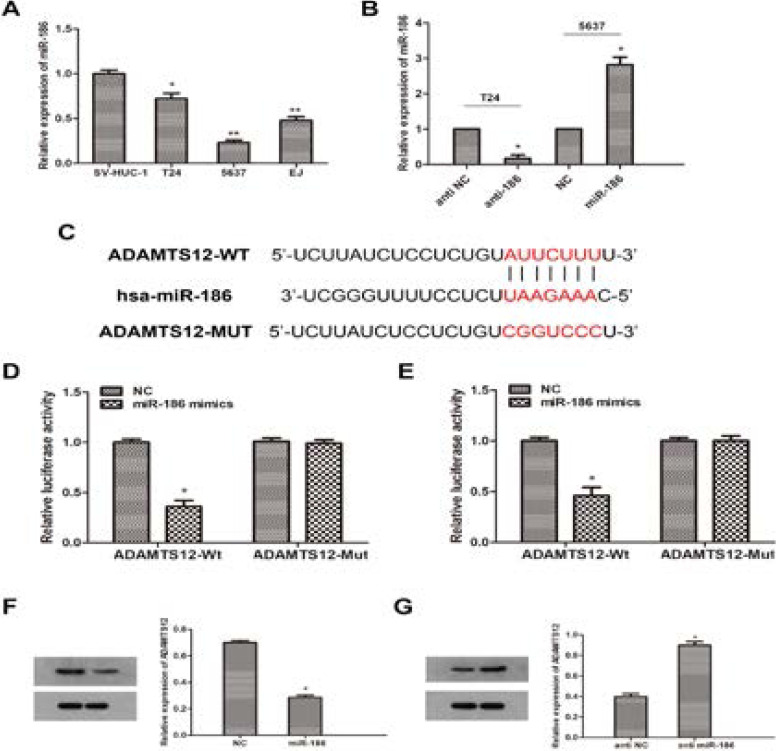
MiR-186 directly regulate ADAMTS12 expression in vitro. (A) Expression of miR-186 in bladder cancer cell lines (T24, 5637 and EJ) and human bladder epithelial cells (SV-HUC-1) (*P<0.05) vs. SV-HUC-1 group) (B) Detection of miR-186 in T24 cells transfected with miR-186 inhibitor and 5637 cells transfected with miR-186 mimics by qRT-PCR (*P<0.05 compared with the corresponding NC group) (C) Sequence alignment of miR-186 binding site predicted in ADAMTS12 3′UTR and its mutation sequence in luciferase reporter gene analysis ( D, E) Luciferase reporter analysis was performed in T24 and 5637 cells co-transfected with miR-186 mimic and a reporter vector containing ADAMTS12 3′UTR or mutated ADAMTS12 3′UTR. The relative activity (F, G) of luciferase was introduced in the Western blot and qRT PCR analysis of ADAMTS12 expression of miR-186 inhibitor transfected in T24 cells and ADAMTS12 expression of miR-186 mimic transfected in 5637 cells (*Compared with the corresponding NC group, P<0.05) (*Compared with the corresponding NC simulation group, P<0.05).

To further investigate the correlation between miR-186 and ADAMTS12, we used normal bladder epithelial cell line (SV-HUC-1) and BCa cell lines (T24, 5637 and EJ) for in vitro study. qRT-PCR results showed that miR-186 was down-regulated to varying degrees in the total three BCa cells compared with normal group (Fig. 2A). Then, we chose T24 and 5637 cells to simulate different stage of cancer, and transfected cells with miR-186 mimics or anti-miR-186 to obtain miR-186 over-expressed or knockdown cells (Fig. 2B). As the data of western blotting revealed, the expression of ADAMTS12 was significantly downregulated in miR-186 overexpressed cells while moderately increased in miR-186 knockdown cells (Fig. 2F, G). Subsequently, in silico prediction indicated that ADAMTS12 was a downstream target of miR-186, and predicted a putative binding site of miR-186 in the 3′UTR of ADAMTS12 (Fig. 2C). Luciferase reporter assays revealed that the luciferase activity was significantly decreased in cells transfected with miR-186 mimics and ADAMTS12-wt, while was not different statistically after transfected with ADAMTS12-MUT (Fig. 2F, G). In summary, these results suggested that miR-186 can negatively regulate the expression of ADAMTS12 by directly binding to ADAMTS12.

### miR-186 modulate the malignant phenotypes of BCa

**Figure 3:**
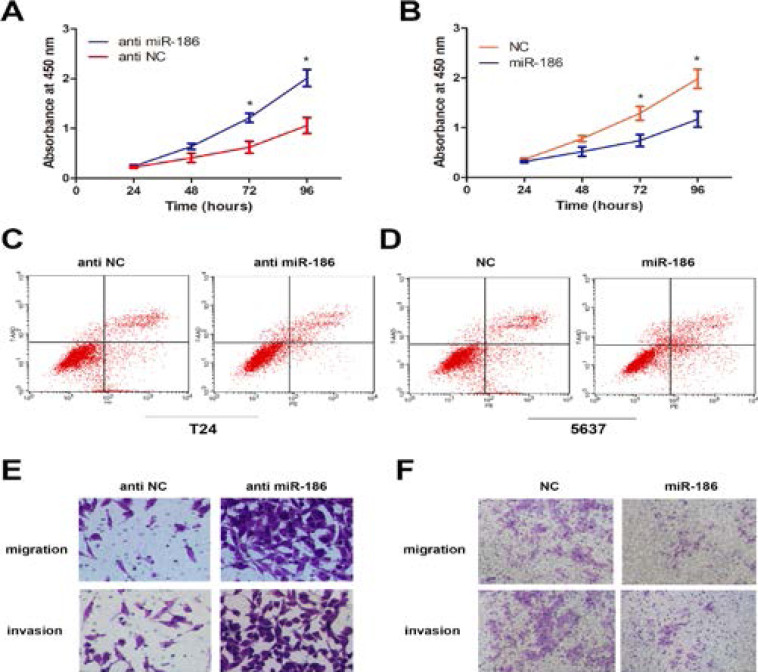
miR-186 regulates malignant phenotypes of BCa in vitro. (A, B) CCK-8 assay was performed to determine the cell viability in BCa cells after transfection. Detect absorbance values at 24, 48, 72, and 96 hours after transfection (*Compared with the corresponding NC group, P<0.05) (C, D) Flow cytometry to detect miR-186 inhibitor-transfected T24 cells and transfected cells Apoptosis of 5637 cells infected with miR-186 mimics (*P<0.05 compared with the corresponding NC group) (E, F) After transfection of T24 and 5637 cells, cross-well migration and invasion experiments were performed to detect cell Migration and invasion capabilities. The number of cells in 5 random areas was counted in units of 200 × magnification (*P<0.05 compared with the corresponding NC group).

In order to clarify the role of miR-186 in the proliferation of BCa cells, CCK-8 analysis was performed. The results showed that compared with the control group, the viability of T24 cells transfected with miR-186 mimics was significantly inhibited, while the viability of T24 cells transfected miR-186 inhibitors was significantly increased (Fig.3 A, B). In addition, we also studied the role of miR-186 in BCa cell apoptosis. Flow cytometry data showed that compared with the control group, overexpression of miR-186 resulted in a significant rate of apoptosis, and this inhibition was eliminated by the use of miR-186 inhibitors (Figure 3 C, D). In addition, we also tested the effect of miR-186 on cell migration and invasion. The results of transwell experiment showed that the number of miR-186 high expression group was significantly lower than that of NC group, while the number of miR-186 knockdown group was significantly increased (Figure 3E, F). In conclusion, our research proves that miR-186 acts as a tumor suppressor in BCa cells.

### Influence of miR-186 on Wnt/β-catenin signaling pathway in vitro.

**Figure 4:**
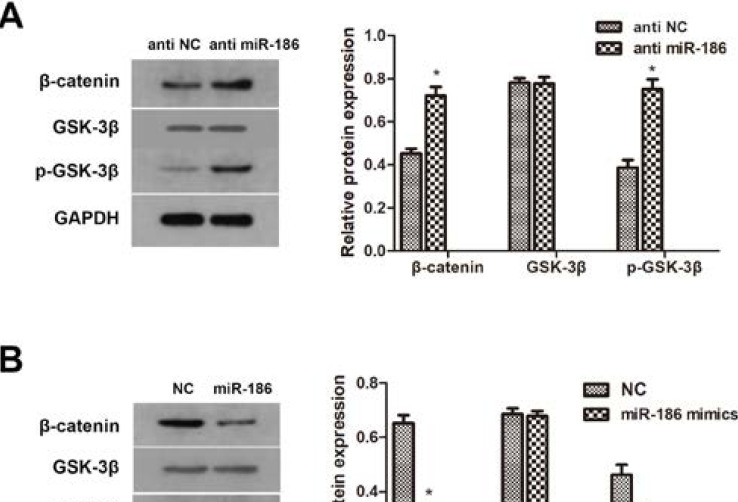
miR-186 involves in the regulation of Wnt/β-catenin signalling pathway. (A, B) Protein level β-catenin, p-GSK-3β, GSK-3β western blot detects T24 cells transfected with miR-186 inhibitor and 5637 cells transfected with miR-186 analog. GAPDH was used as an internal control (*P<0.05 compared with the corresponding NC group).

In order to study the role of miR-186 in the regulation of Wnt/β-catenin signalling pathway, we analyzed the phosphorylation level of GSK-3β and β-catenin. Western blotting showed that miR-186 overexpression significantly induced the phosphorylation of GSK-3β and β-catenin. The total level of GSK-3β remain the same related to the expression of miR-186. (Fig. 4 A, B). Taken together, these results suggest that miR-186 may be an important regulator of the Wnt/β-catenin signalling pathway, which may be an important factor to the tumorigenesis of BCa cells.

## Discussion

Bladder cancer is a highly malignant and rapidly progressing cancer. Recent studies have revealed the prognostic outcomes of patients with BCa, particularly MIBC, with only 5% and 48% 5-year overall survival (OS) reported for untreated and treated groups, respectively 21. A large number of recent reports indicate that functional miRNA expression models and variation are critical in bladder carcinoma 22. The current study revealed that abnormal expression of miR-186 regulated the progression of BCa, our study showed that compared with the normal control group, the expression of miR-186 was significantly down-regulated in BCa cells and clinical samples. Function assays revealed that miR-186 overexpression can repressproliferation and invasion in vitro, suggesting that miR-186 acts as a tumor suppressor in BCa.

ADAMTS12 is a metalloprotease complex of the ADAMTS family, which contains multiple Thrombospondin-1 repeats 23. It has been found to be involved in the regulation of variety of biological functions, such as inflammation, angiogenesis and organogenesis^24^. Up to now, a large number of researches have observed that ADAMTS metalloproteinase is involved in various physiological pathological processes of cancers. According to the current findings, ADAMTS12 exhibits dual pro- and/or anti-tumor effects in proteolytic or non-proteolytic pathways. In renal cell carcinoma, ADAMTS12 is listed as one of the seven extracellular matrix (ECM) genes, and the up-regulation and high expression of ADAMTS12 may contribute to metastasis^25^. With the enhancement of tumor invasiveness of the ADAMTS12 high-expressing cell line, the tumor-promoting function of human choriocarcinoma JEG-3 cells has also been confirmed^26^. In contrast, some results of in vitro and in vivo studies indicate that ADAMTS12 deficiency enhanced angiogenesis and tumor invasiveness, indicating that ADAMTS12 is a potential anti-tumor factor^27^. However, the specific role and mechanism of ADAMTS12 in BCa have not been fully elucidated. Our study identified ADAMTS12 as a direct downstream target of miR-186.

Wnt

In short, miR-186 expression is closely correlated to the progression of BCa. The in vitro study identified miR-186 as a tumor suppressor in BCa by binding to ADAMTS12. Thus, this miRNA has a potential to become a promising therapeutic target for BCa.

## Declarations

### Availability of data and material

The datasets used and analysed during the current study are available from the corresponding author on reasonable request.
